# Danish National Penile Cancer Database, DaPeCa-data: characteristics, management, and survival 2000–2025

**DOI:** 10.1007/s11255-026-05121-4

**Published:** 2026-04-06

**Authors:** Janni Mølsted, Anne Birgitte Als, Jørgen Bjerggaard Jensen, Mikael Aagaard, Jakob Kristian Jakobsen

**Affiliations:** 1 Department of Clinical Medicine, Aarhus University, Aarhus, Denmark; 2 Department of Urology, Aarhus University Hospital, Aarhus, Denmark; 3 Department of Oncology, Aarhus University Hospital, Aarhus, Denmark; 4 Department of Urology, Copenhagen University Hospital, Copenhagen, Denmark

**Keywords:** Penile cancer, Database, National, Sentinel node, Surgical treatment, Survival

## Abstract

**Purpose:**

To present the characteristics of Danish penile cancer patients, treatment strategies, and prognosis.

**Methods:**

The Danish National Penile Cancer database currently contains information on 1,397 patients with penile cancer referred to a university center from 2000 to 2025. The database was established by retrospective medical chart review. The set of variables were defined by the Danish Multidisciplinary Cancer Group, DaPeCa.

**Key findings:**

Between 2000 and 2025, 1,397 patients with penile cancer were evaluated at a tertiary Danish center; 1,200 (86%) were treated with curative intent. Most patients presented with pT1-2 disease (79%), while 206 (15%) had pN3 disease and 47 (3.4%) had distant metastases at diagnosis. Surgical nodal staging was performed in 87% of patients. Organ-preserving treatment was used in 47% of curatively treated patients and in 75% of pT1 tumors. The overall 5-year cumulative incidence (CI) of penile cancer–specific death was 16% (95% CI 14–19), compared with 11% (95% CI 8.8–13) among curatively treated patients and 53% (95% CI 45–60) in non-curative cases. Mortality increased markedly with disease stage, reaching a CI of 58% in AJCC stage IV and 83% in patients with distant metastases. In multivariable analyses, higher T stage and nodal stage were the strongest predictors of disease-specific survival.

**Conclusion:**

45% of patients were diagnosed with pT1 tumors, 27% presented with nodal metastases (pN +), and 3.4% had distant metastases at diagnosis. Overall 5-year cumulative incidence of penile cancer-specific death was 16% (14%, 19%). Most patients were treated with curative intent and achieved favorable survival outcomes. T stage and nodal stage were the strongest independent predictors of disease-specific survival.

## Introduction

Penile cancer is a rare malignancy associated with substantial morbidity and impact on quality of life. Despite its low incidence, the clinical burden remains considerable, and comprehensive data are limited as a consequence of the rarity of the disease. To address this, the Danish National Penile Cancer database (DaPeCa-data) was established in 2011 by the Danish Penile Cancer Group. The database has generated several publications and has recently been migrated to a permanent electronic platform and expanded with additional variables, positioning it among the largest contemporary national datasets on penile cancer.

The aim of the present study is to present this updated database and present data on the patient cohort, tumor characteristics, management, and survival.

## Patients and methods

The DaPeCa-data was established and is maintained by retrospective chart and pathology report review. Patients evaluated for penile cancer at highly specialized centers in Denmark between 01–01-2000 and 01–01-2026 were included. The number of specialized centers was five from 2000–2008 and two from 2009 until today. Authors and investigators had access to patients’ records from the electronic patient chart systems in the Central Denmark Region (EPJ) and The Capitol Region of Denmark (SundhedsPlatformen/Epic). The DaPeCa-data database contains more than 150 variables per patient encompassing key clinical milestones, behavioral markers, patient characteristics, tumor characteristics according to the TNM classification, histopathology, primary tumor treatment, oncological treatment, lymph node management, treatment-related complications, recurrence, survival, and follow-up data. Penile cancer found only at postmortem autopsy and penile cancer patients who were never referred to a university center are not included.

Patients with only Penile Intraepithelial Neoplasia (PeIN, previously referred to as pTis/Carcinoma in Situ) without synchronous penile cancer are not included. Vital status for this manuscript was determined on 01–01-2026.

TNM status in the database is evaluated and updated according to International Collaboration on Cancer Reporting (ICCR) TNM8 [1]. Penile cancer stages were also classified according to the American Joint Committee on Cancer (AJCC) 8th edition protocol [2], which incorporates TNM stage, histological grade, and the presence of perineural or vascular invasion into composite stages I–IV. AJCC stage was assigned retrospectively based on variables recorded in the database. Patients with missing data required for complete staging were classified as stage X (stX). Histopathological grade of the tumors was evaluated according to the WHO/ISUP tri-part system [1]. As HPV-typing has not been standard procedure in penile cancer pathology in Denmark, Cyclin-dependent Kinase Inhibitor protein p16^INK4a^ (P16) has been used in recent periods on most patients as part of imnunostaging, as P16 positivity is established as a predictor for presence of high-risk HPV [3, 4]. Cause of death, if not recorded in the available medical charts, was classified as a penile cancer-related death if a deceased patient had evidence of untreated primary penile cancer, recurrent disease, or metastasis at the time of death. The time of death is updated to the medical chart systems once every 24 h from the Danish Cause of Death registry.

Organ-preserving treatment (OPT) refers to the penile surgical treatment modalities that leave a patient with a near-intact penis. In this article, glansectomy is not included as OPT, as it is registered as partial penectomy. If positive margins are identified after penile tumor surgery, re-operation is performed to achieve negative margins. Primary surgery defines all surgeries performed as initial treatment. For all Danish clinically node negative (cN0) penile cancer patients subject to lymph node diagnostics since 2000, inguinal sentinel node biopsy (SNB) has been used as the initial lymph node diagnostic procedure [5]. In cases with overt suspicion of inguinal lymph node metastases (clinical, radiological or needle biopsy-verified) (cN +), primary inguinal lymphadenectomy was performed. If extranodal extension or metastatic pelvic lymph nodes are identified (pN3), the case is discussed at a multidisciplinary team conference involving penile cancer-specialized urologists, oncologists, pathologists, and radiologists. At this meeting, the oncological treatment strategy is determined, including consideration of external beam radiation therapy (EBRT), chemotherapy, or combined chemoradiation.

When analyzing histopathological subtypes, mixed tumors (i.e., those containing more than one subtype) are classified by assigning them to a single category according to the most aggressive subtype in the order: clear cell > sarcomatoid > basaloid > scc > warty > verrucous > papillary. Survival estimates are calculated using the Kaplan–Meier (KM) method, cumulative incidence (CI) calculations using the Aalen–Johansen method. Multivariable survival analysis was performed using Cox proportional hazards regression with the Efron method for tied event times. Models were fitted separately for disease-specific survival (DSS) in the curative-intent cohort. The proportional hazards assumption was assessed for each covariate individually using Schoenfeld residuals with rank transformation. Results are presented as hazard ratios (HR) with 95% confidence intervals. Statistical significance was set at *p* < 0.05.

The study was approved by relevant local authorities, and the approval number is 1–10-72–150-22. First and last authors have full access to all the data in the study and take responsibility for the integrity of the data and the accuracy of the analyses. The database is under the auspices of the DaPeCa group.

## Results

In the inclusion period between 01-01-2000 and 01-01-2025, 1,397 patients were diagnosed with penile cancer and evaluated at a tertiary center in Denmark.

### Patient and tumor characteristics

Patient and primary tumor characteristics of the cohort are presented in Table 1.

**Table 1:** Penile cancer patient and tumor characteristics, 2000–2025

Time period	2000–2025	2000–2009	2010–2017	2018–2025
Characteristic	*n* = 1397	*n* = 371	*n* = 482	*n* = 544
Treatment strategy				
Curative	1,200 (86%)	327 (88%)	412 (85%)	461 (85%)
Non-curative	197 (14%)	44 (12%)	70 (15%)	83 (15%)
Age group				
< 40 years	22 (1.6%)	8 (2.2%)	6 (1.2%)	8 (1.5%)
40–59 years	291 (21%)	96 (26%)	95 (20%)	100 (18%)
60–79 years	811 (58%)	218 (59%)	277 (57%)	316 (58%)
80 + years	273 (20%)	49 (13%)	104 (22%)	120 (22%)
Age at diagnosis (median (Q1, Q3))	68 (60–77)	65 (58–73)	69 (61–77)	71 (62–79)
Smoking status				
Never	518 (37%)	149 (40%)	143 (30%)	226 (42%)
Previous	370 (26%)	41 (11%)	164 (34%)	165 (30%)
Current	433 (31%)	177 (48%)	135 (28%)	121 (22%)
No data	76 (5.4%)	4 (1.1%)	40 (8.3%)	32 (5.9%)
Living arrangement				
Own house	900 (64%)	220 (59%)	312 (65%)	368 (68%)
Apartment/rental	386 (28%)	129 (35%)	120 (25%)	137 (25%)
Institution	66 (4.7%)	21 (5.7%)	19 (3.9%)	26 (4.8%)
No data	45 (3.2%)	1 (0.3%)	31 (6.4%)	13 (2.4%)
Civil status				
Cohabiting/married	917 (66%)	254 (68%)	312 (65%)	351 (65%)
Living alone	415 (30%)	115 (31%)	138 (29%)	162 (30%)
No data	65 (4.7%)	2 (0.5%)	32 (6.6%)	31 (5.7%)
CCI at diagnosis—groups				
< 2	246 (18%)	95 (26%)	76 (16%)	75 (14%)
2–4	793 (57%)	216 (58%)	277 (57%)	300 (55%)
5–8	301 (22%)	51 (14%)	93 (19%)	157 (29%)
9 +	32 (2.3%)	9 (2.4%)	11 (2.3%)	12 (2.2%)
No data	25 (1.8%)	0 (0%)	25 (5.2%)	0 (0%)
CCI at diagnosis (median (Q1, Q3))	3.00 (2.00–4.00)	2.00 (1.00–4.00)	3.00 (2.00–4.00)	4.00 (2.00–5.00)
T stage				
pT1	626 (45%)	194 (52%)	203 (42%)	229 (42%)
pT2	475 (34%)	107 (29%)	167 (35%)	201 (37%)
pT3	241 (17%)	55 (15%)	94 (20%)	92 (17%)
pT4	30 (2.1%)	8 (2.2%)	13 (2.7%)	9 (1.7%)
pTx	25 (1.8%)	7 (1.9%)	5 (1.0%)	13 (2.4%)
N stage				
pN0	833 (60%)	227 (61%)	295 (61%)	311 (57%)
pN1	131 (9.4%)	33 (8.9%)	42 (8.7%)	56 (10%)
pN2	48 (3.4%)	13 (3.5%)	19 (3.9%)	16 (2.9%)
pN3	206 (15%)	40 (11%)	82 (17%)	84 (15%)
pNx	179 (13%)	58 (16%)	44 (9.1%)	77 (14%)
M stage				
M0	1,230 (88%)	334 (90%)	421 (87%)	475 (87%)
M1	47 (3.4%)	12 (3.2%)	12 (2.5%)	23 (4.2%)
Mx	120 (8.6%)	25 (6.7%)	49 (10%)	46 (8.5%)
Grade				
G1	441 (32%)	166 (45%)	168 (35%)	107 (20%)
G2	514 (37%)	145 (39%)	182 (38%)	187 (34%)
G3	430 (31%)	60 (16%)	132 (27%)	238 (44%)
Gx	12 (0.9%)	0 (0%)	0 (0%)	12 (2.2%)
AJCC stage				
I	406 (29%)	147 (40%)	136 (28%)	123 (23%)
II	568 (41%)	125 (34%)	197 (41%)	246 (45%)
III	172 (12%)	46 (12%)	57 (12%)	69 (13%)
IV	235 (17%)	46 (12%)	89 (18%)	100 (18%)
stX	16 (1.1%)	7 (1.9%)	3 (0.6%)	6 (1.1%)
Histological subtype^§^				
SCC	962 (69%)	281 (76%)	346 (72%)	335 (62%)
Basaloid	211 (15%)	15 (4.0%)	68 (14%)	128 (24%)
Verrucous	94 (6.7%)	37 (10.0%)	36 (7.5%)	21 (3.9%)
Warty	40 (2.9%)	2 (0.5%)	10 (2.1%)	28 (5.1%)
Sarcomatoid	25 (1.8%)	4 (1.1%)	8 (1.7%)	13 (2.4%)
Papillary	22 (1.6%)	14 (3.8%)	3 (0.6%)	5 (0.9%)
Clear cell	12 (0.9%)	0 (0%)	8 (1.7%)	4 (0.7%)
Other	5 (0.4%)	1 (0.3%)	2 (0.4%)	2 (0.4%)
No data	26 (1.9%)	17 (4.6%)	1 (0.2%)	8 (1.5%)
*Mixed tumors*^*¶*^	*148 (11%)*	*3 (0.8%)*	*44 (9.1%)*	*101 (19%)*
p16 overexpression				
Yes	347 (25%)	2 (0.5%)	114 (24%)	231 (42%)
No	299 (21%)	0 (0%)	76 (16%)	223 (41%)
No data	751 (54%)	369 (99%)	292 (61%)	90 (17%)
HPV status				
Positive	52 (3.7%)	0 (0%)	4 (0.8%)	48 (8.8%)
Negative	78 (5.6%)	0 (0%)	28 (5.8%)	50 (9.2%)
Not performed/No data	1,267 (91%)	371 (100%)	450 (93%)	446 (82%)

*BMI* body mass index

*CCI* Charlson Comorbidity Index

TNM staging according to DMCG guidelines on Penile cancer pathology, version 2.2

AJCC staging according to American Joint Committee on Cancer (AJCC) 8th edition protocol

T stage: primary tumor stage, p: pathology

N stage: lymph node stage, p: pathology

M stage: distant metastases

Grade: histopathological grade

SCC: squamous cell carcinoma

Gx: grade not specified

stX: no AJCC stage

P16: Cyclin-dependent Kinase Inhibitor protein p16^INK4a^

HPV: Human Papillomavirus

^§^If mixed type tumor, subtype classified as most aggressive component subtype: Clear Cell > Sarcomatoid > Basaloid > SCC > Warty > Verrucous > Papillary

^¶^Number of mixed tumors as an individual subgroup irrespective of the component subtypes

A total of 626 patients (45%) presented with pT1 tumor, 475 (34%) presented with pT2, 241 (17%) with pT3 and 30 (2.1%) with pT4. For 25 (1.8%), the T stage was unknown (pTx).

For 209 patients (15%), margin status after primary penile surgery was positive or uncertain. Among these, 30% had locally advanced disease and proceeded to oncological treatment, and 20% were managed in a non-curative setting.

In the cohort, 87% of patients were surgically staged for nodal metastases during the initial phase of treatment, 833 (60%) presented as pN0 without initial lymph node metastases, 131 (9.4%) with pN1, 48 (3.4%) with pN2 and 206 (15%) with pN3 disease.

Distant metastases (M +) were diagnosed in 47 patients (3.4%) at the time of diagnosis, 120 (8.6%) could not be assessed for distant metastases (Mx).

Of 1,397 patients, 833 (60%) had formal nodal staging (pN0) and 179 (13%) had undetermined nodal status (pNx). Compared to the overall cohort, pNx patients were older (median 79 vs 68 years), had higher comorbidity burden (median CCI 4 vs 3), and worse performance status (PS ≥ 2: 46% vs 13%). While 86% of the overall cohort received curative-intent treatment, 68% of pNx patients had a palliative treatment strategy. pNx patients also presented with more advanced disease than the overall cohort, with higher rates of pT3/4 + tumors (39% vs 21%), M1 or Mx status (50% vs 12%), and more frequently underwent total penectomy or no surgical treatment (34% vs 16%). After multivariable adjustment, pNx status was not an independent predictor of disease-specific survival compared to pN0 (HR 1.09, 95% CI 0.33–3.53, *p* = 0.892).

**Fig. 1:**
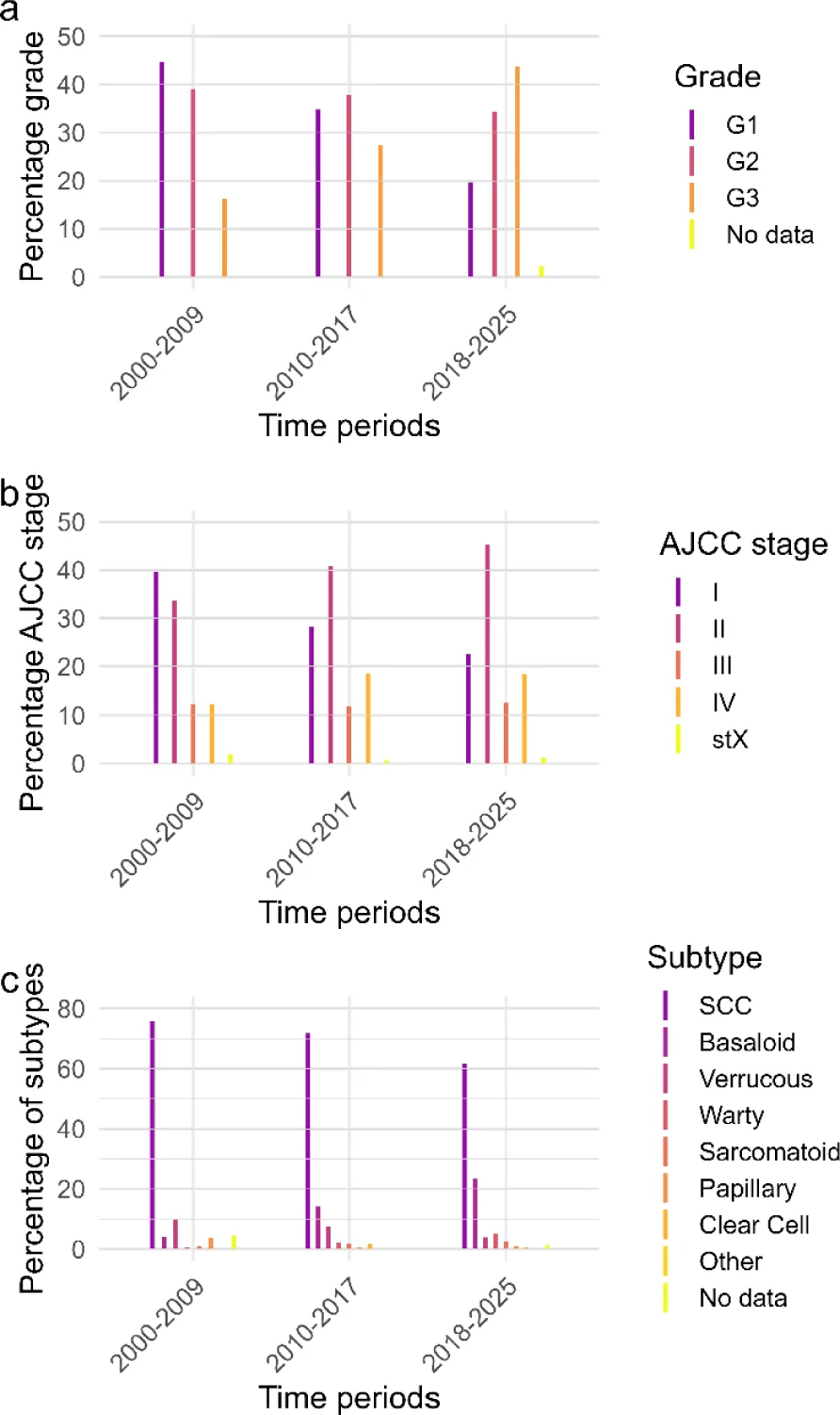
Grade, AJCC stages, and histopathological subtypes over time: **a** tumor grade, **b** AJCC stage, **c** histopathological subtype of penile cancer at diagnosis across time periods: 2000–2009 *n* = 371, 2010–2017 *n* = 482, and 2018–2025 *n* = 544. *AJCC* American Joint Committee on Cancer, *SCC* squamous cell carcinoma, *Gx* grade not specified, *stX* no AJCC stage. If mixed type tumor, subtype classified as most aggressive component subtype: clear cell > sarcomatoid > basaloid > SCC > warty > verrucous > papillary. Grade: histopathological grade of the tumors according to the WHO/ISUP tri-part system based on nuclear polymorphia and keratine production

Histopathological grade of the tumors shifted throughout the time periods. Between 2000 and 2009, G1 tumors accounted for 45% of cases, whereas G3 tumors represented only 16%. In 2017–2025, G1 tumors were observed in 20% of cases, and G3 tumors in 44%. G2 tumor prevalence remained relatively unchanged throughout the time periods at 34–39% (Fig. 1a).

AJCC stage II disease was most common (41%), followed by stage I (29%), stage IV (17%), and stage III (12%). Over time, a notable stage shift was observed: stage I cases declined by nearly half, from 40% in 2000–2009 to 23% in the most recent period, while stage II increased from 34 to 45%. Stage III remained stable at 12–13%, whereas stage IV rose from 12 to 18% (Fig. 1b). The histopathological subtype prevalences of penile cancer are presented in Fig. 1c, demonstrating a marked increase in the prevalence of the basaloid subtype (from 4 to 24%) across the study periods, alongside a decline in squamous cell carcinoma (SCC) prevalence (from 76 to 62%).

### Treatment

Eighty-six percent of the study cohort was treated initially with curative intent and for 197 patients (14.0%), the initial treatment strategy was non-guideline driven, with non-curative intent or palliative.

Treatment modalities for the primary penile tumor are presented in Table 2.

**Table 2:** Primary penile tumor treatment modality, 2000–2025

	2000–2025	2000–2009	2010–2017	2018–2025
	*n* = 1397	*n* = 371	*n* = 482	*n* = 544
Treatment modality				
Biopsy only^†^	22 (1.6%)	2 (0.5%)	4 (0.8%)	16 (2.9%)
Circumcision only	89 (6.4%)	31 (8.4%)	21 (4.4%)	37 (6.8%)
Local resection ± laser	451 (32.3%)	130 (35.0%)	176 (36.5%)	145 (26.7%)
Partial penectomy (including glansectomy)	506 (36.2%)	111 (29.9%)	163 (33.8%)	232 (42.6%)
Total penectomy	114 (8.2%)	40 (10.8%)	48 (10.0%)	26 (4.8%)
Oncological treatment of penile tumor	18 (1.3%)	13 (3.5%)	0 (0.0%)	5 (0.9%)
Non-curative strategy	197 (14.1%)	44 (11.9%)	70 (14.5%)	83 (15.3%)

**Fig. 2:**
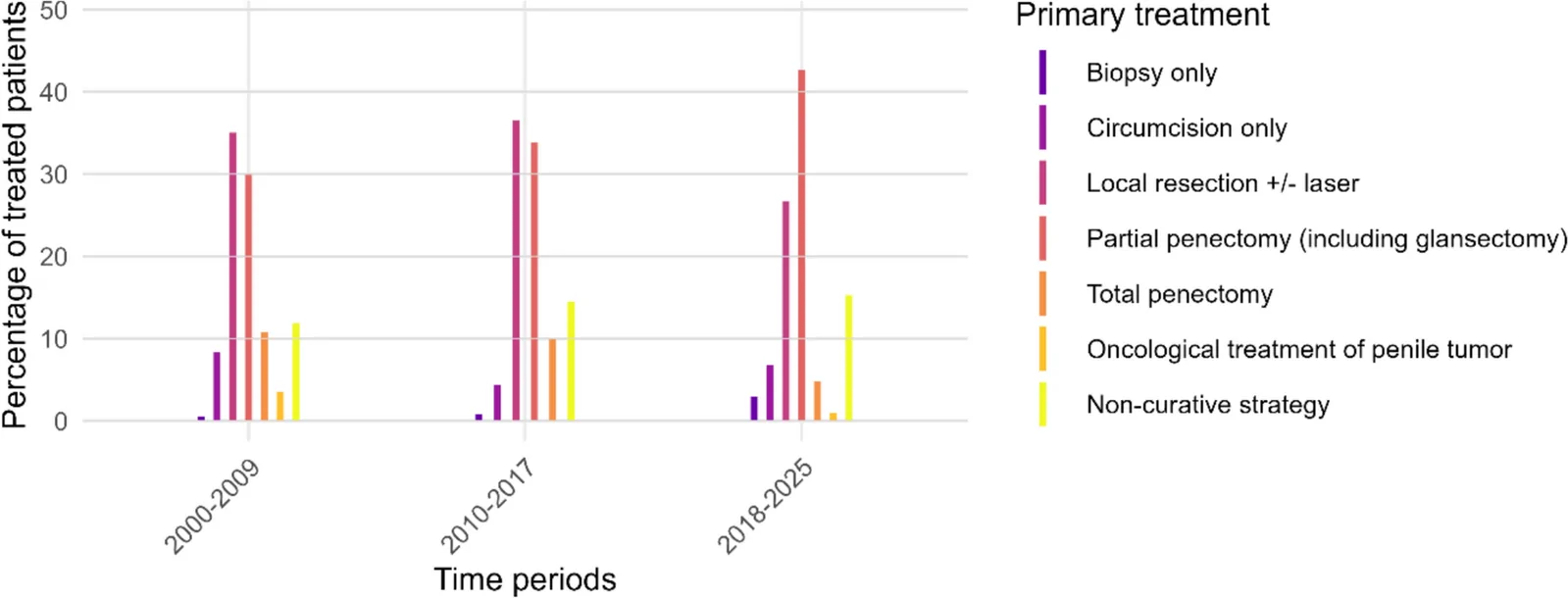
Primary treatment modality for penile cancer patients (percentage) distributed across time periods: 2000–2009 *n* = 371, 2010–2017 *n* = 482, and 2018–2025 *n* = 544

We observed an overall development in treatment strategies towards less total penectomies and more partial penectomies (Fig. 2).

More patients received initial non-curative treatment in recent years compared to earlier. Of all 1.200 patients with an initial curative treatment strategy, 441 (75%) patients out of the 586 with pT1 tumors and 100 (23%) patients of the 429 with T2 tumors were treated with OPT (Table 3).

**Table 3:** Organ-preserving treatment as primary tumor treatment modality: only patients with initial curative treatment strategy *n* = 1.200

	2000–2025 *n* = 1200		2000–2009 *n* = 327		2010–2017 *n* = 412		2018–2025 *n* = 461	
T stage	n	OPT (%)^‡^	n	OPT (%)^‡^	n	OPT (%)^‡^	n	OPT (%)^‡^
T1	587	441 (75.1%)	181	135 (74.6%)	192	151 (78.6%)	214	155 (72.4%)
T2	428	100 (23.4%)	98	17 (17.3%)	145	44 (30.3%)	185	39 (21.1%)
T3	161	8 (5%)	39	3 (7.7%)	68	4 (5.9%)	54	1 (1.9%)
T4	13	2 (15.4%)	2	1 (50%)	6	1 (16.7%)	5	0 (0%)
Tx	11	11 (100%)	7	7 (100%)	1	1 (100%)	3	3 (100%)
Sum	1200	562 (46.8%)	327	163 (49.8%)	412	201 (48.8%)	461	198 (43%)

^†^ ‘Biopsy only’ refers to patients where initial biopsy removed the cancer completely

^‡^ Patients in specific T stage who received OPT (% of T stage group who received OPT)

Organ-preserving treatment (OPT): biopsy only, circumcision only, local resection with and without laser ablation

Non-organ preserving treatment: no treatment, partial penectomy incl. glansectomy, total penectomy, primary oncological treatment

As part of primary treatment and staging, 141 patients (11%) underwent primary unilateral sentinel node biopsy, and 852 (61%) underwent primary bilateral sentinel node biopsy. Lymphadenectomy was performed unilaterally on 130 (9%) and bilaterally on 95 (7%) patients as part of the primary treatment and diagnosis, either as a result of a positive sentinel node procedure, overt imaging, or clinical suspicion confirmed by positive ultrasound-guided inguinal lymph node biopsy (either fine needle aspiration cytology or coarse needle biopsy).

**Fig. 3:**
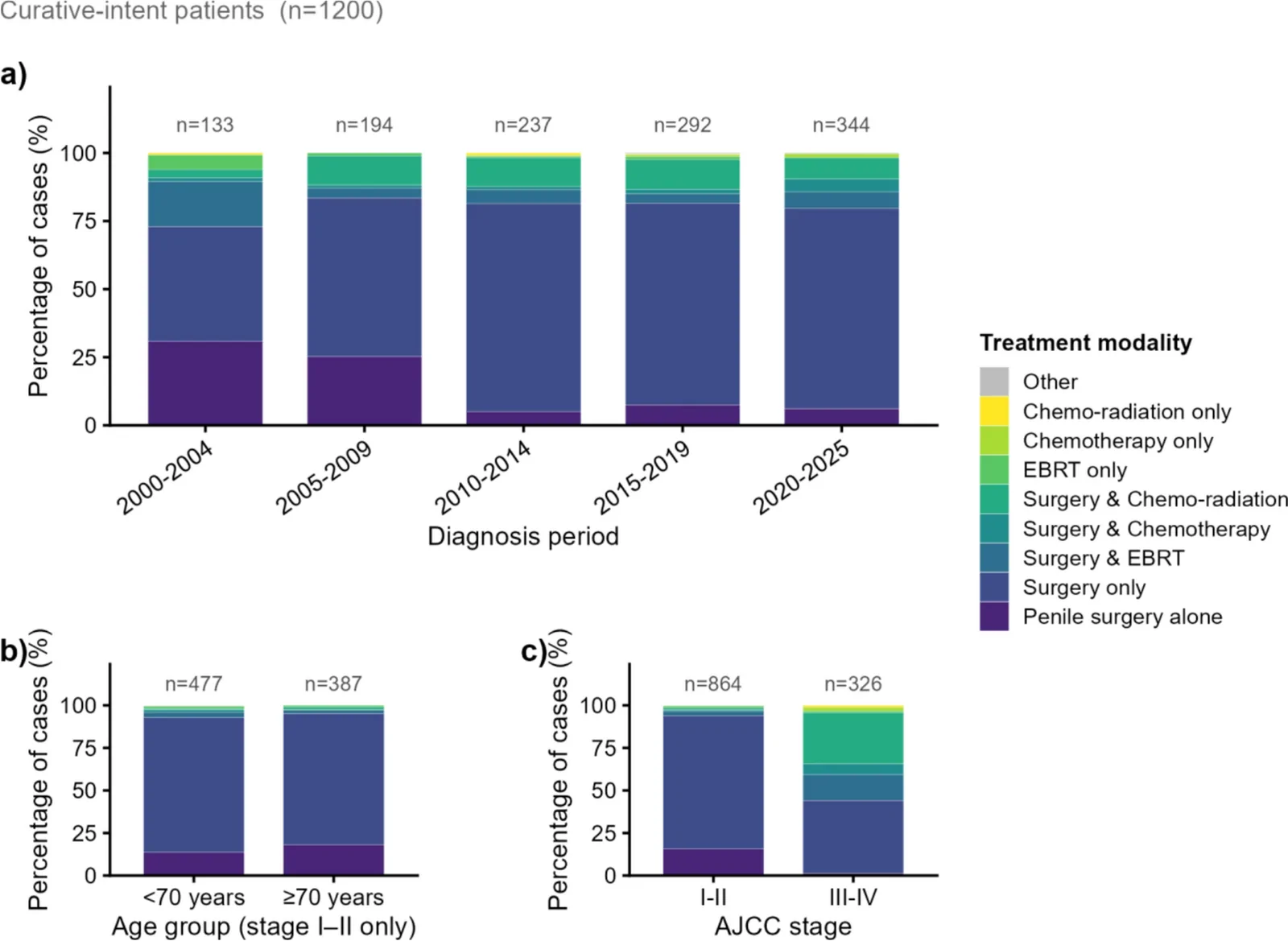
Treatment modalities in curative-intent penile cancer patients, 2000–2025. Stacked bar charts illustrating the distribution of treatment modalities among curative-intent patients by diagnosis period (**a**), age group in AJCC stage I–II disease (**b**), and AJCC stage (**c**). “Penile surgery alone” denotes patients who received primary penile surgery without lymph node surgery or oncological treatment, “Surgery alone” denotes patients who received both penile and lymph node surgery. All other categories reflect lymph node management—sentinel node biopsy, inguinal lymph node dissection, or pelvic lymph node dissection—with or without first-line oncological treatment. Oncological treatment categories reflect first-line modality and may include treatment administered in the setting of recurrent disease. The total number of patients in each subgroup is shown above each bar. EBRT: external beam radiotherapy; AJCC: American Joint Committee on Cancer staging system, 8th edition

The majority of curatively treated patients received penile and lymph node surgery without oncological treatment (964 patients, 80%). The surgery alone group increased from 42% in 2000–2004 to 73–76% from 2010 onwards (Fig. 3a). Penile surgery alone decreased from 30% to 5–6% over the same period. Among patients receiving oncological treatment alongside surgery, a shift from surgery and EBRT (17% in 2000–2004) towards surgery and chemoradiation (11% in 2015–2019) was observed.

No meaningful difference in treatment modality was observed between patients aged under and over 70 years in stage I–II disease (Fig. 3b). Treatment differed markedly by stage (Fig. 3c): 94% of stage I–II patients received surgery alone compared to 44% of stage III–IV patients, of whom 30% received surgery combined with chemoradiation and 4% received oncological treatment without lymph node surgery.

### Survival

Survival estimates can be seen in Table 4.

**Table 4:** Survival estimates penile cancer patients in cohort *n* = 1397 2000–2025

Variable	Subgroup	*n*	1-year KM survival estimate, % (95% CI)	5-year KM survival estimate, % (95% CI)	5-year cumulative incidence (96% CI)
Overall		1.397	91% (90%, 93%)	83% (80%, 85%)	16% (14%, 19%)
Curative strategy		1.200	95% (94%, 96%)	89% (87%, 91%)	11% (8.8%, 13%)
Non-curative strategy		197	65% (58%, 72%)	36% (29%, 46%)	53% (45%, 60%)
Age					
	< 40 years	22	95% (87%, 100%)	91% (80%, 100%)	9.1% (1.5%, 26%)
	40–59 years	291	95% (92%, 98%)	86% (81%, 90%)	14% (10%, 19%)
	60–79 years	811	92% (90%, 94%)	84% (81%, 87%)	16% (13%, 18%)
	80 + years	273	84% (79%, 89%)	74% (67%, 80%)	23% (18%, 28%)
pT stage					
	pT1	626	99% (98%, 100%)	96% (95%, 98%)	3.5% (2.2%, 5.3%)
	pT2	475	92% (90%, 95%)	84% (80%, 87%)	15% (12%, 19%)
	pT3	241	78% (72%, 83%)	56% (49%, 63%)	41% (35%, 48%)
	pT4	30	39% (24%, 62%)	5.9% (1.0%, 34%)	85% (61%, 95%)
	pTx	25	59% (41%, 84%)	48% (31%, 76%)	47% (25%, 66%)
pN stage					
	pN0	833	99% (98%, 100%)	96% (94%, 97%)	4.2% (2.9%, 5.8%)
	pN1	131	94% (90%, 98%)	82% (75%, 90%)	16% (10%, 24%)
	pN2	48	91% (83%, 100%)	81% (69%, 94%)	18% (8.2%, 30%)
	pN3	206	68% (61%, 74%)	42% (35%, 50%)	55% (47%, 61%)
	pNx	179	80% (74%, 87%)	64% (56%, 73%)	30% (23%, 37%)
M stage					
	M0	1.230	94% (93%, 95%)	86% (84%, 88%)	13% (11%, 15%)
	M1	47	42% (29%, 60%)	12% (4.7%, 30%)	83% (66%, 92%)
	Mx	120	81% (73%, 89%)	70% (60%, 81%)	25% (17%, 33%)
Grade					
	G1	441	94% (92%, 96%)	90% (87%, 93%)	9.3% (6.8%, 12%)
	G2	514	93% (91%, 95%)	85% (82%, 89%)	14% (11%, 17%)
	G3	430	86% (83%, 90%)	71% (67%, 76%)	27% (23%, 32%)
	Gx	12	79% (56%, 100%)	68% (43%, 100%)	27% (5.6%, 55%)
AJCC stage I–IV					
	I	406	100% (99%, 100%)	98% (97%, 100%)	1.6% (0.68%, 3.4%)
	II	568	96% (94%, 97%)	89% (86%, 92%)	10% (7.6%, 13%)
	III	172	93% (89%, 97%)	82% (76%, 89%)	16% (11%, 22%)
	IV	235	65% (59%, 72%)	39% (32%, 46%)	58% (51%, 64%)
	stX	16	85% (68%, 100%)	77% (56%, 100%)	20% (4.5%, 44%)
Histological subtype^†^					
	SCC	962	91% (89%, 93%)	83% (80%, 86%)	16% (14%, 19%)
	Basaloid	211	95% (92%, 98%)	81% (75%, 88%)	17% (12%, 23%)
	Verrucous	94	96% (92%, 100%)	91% (85%, 97%)	8.8% (4.1%, 16%)
	Warty	40	97% (91%, 100%)	94% (86%, 100%)	5.7% (1.0%, 17%)
	Sarcomatoid	25	76% (61%, 95%)	62% (43%, 90%)	36% (16%, 57%)
	Papillary	22	95% (87%, 100%)	95% (87%, 100%)	4.8% (0.30%, 20%)
	Clear cell	12	64% (41%, 100%)	44% (22%, 88%)	54% (20%, 79%)
	Other	5	80% (52%, 100%)	80% (52%, 100%)	20% (0.43%, 62%)
	No data	26	75% (59%, 95%)	50% (32%, 77%)	42% (23%, 61%)
	*Mixed*^‡^	*148*	94% (89%, 98%)	83% (76%, 91%)	16% (9.3%, 23%)

KM: survival estimates calculated using the Kaplan–Meier method

TNM staging according to DMCG guidelines on Penile cancer pathology, version 2.2

AJCC staging according to American Joint Committee on Cancer (AJCC) 8th edition protocol

T stage: primary tumor stage, p: pathology

N stage: lymph node stage, p: pathology

M stage: distant metastases

Grade: histopathological grade

SCC: squamous cell carcinoma

Gx = grade not specified

stX: no AJCC stage

^†^If mixed type tymor, subtype classified as most aggressive component subtype: clear cell > sarcomatoid > basaloid > SCC > warty > verrucous > papillary

^‡^Mixed tumors as an individual subgroup disregarding the component subtypes

**Fig. 4:**
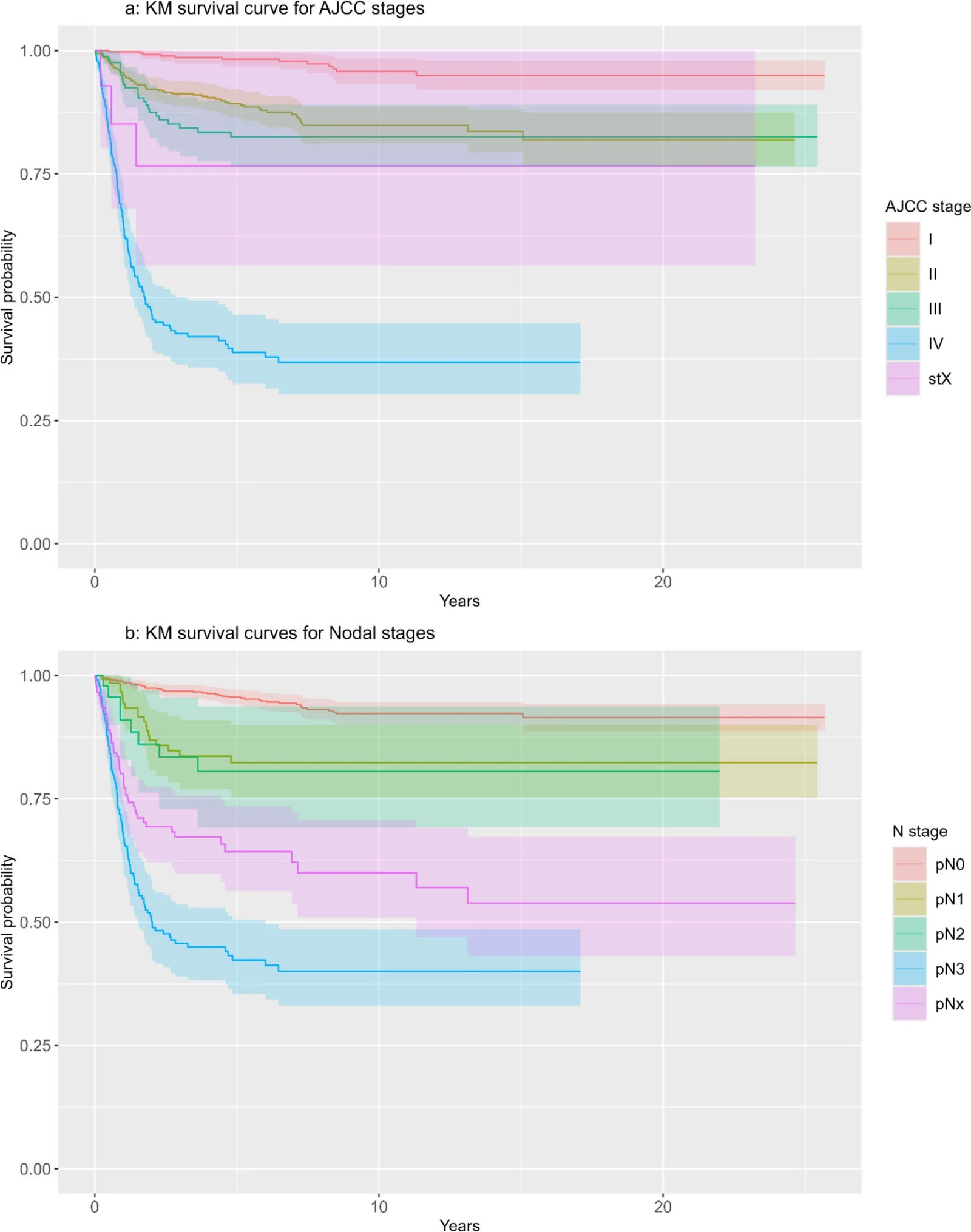
Kaplan–Meier survival curves for **a** AJCC stage and **b** nodal status. *KM* Kaplan–Meier, *AJCC* American Joint Committee on Cancer 8th edition protocol, *stX* no AJCC stage, *N stage* lymph node stage, *p* pathology

Overall, the 5-year cumulative incidence of penile cancer-specific death was 16% (95% CI 14–19). Among patients treated with curative intent, the 5-year cumulative incidence was 11% (95% CI 8.8–13), compared with 53% (95% CI 45–60) in those managed non-curatively. Mortality increased with advancing disease stage: the 5-year cumulative incidence was 1.6% for AJCC stage I, 10% for stage II, 16% for stage III, and 58% for stage IV (Fig. 4a).

Similarly, the 5-year cumulative incidence of penile cancer-specific death was 4.2% for pN0 disease, 16–18% for pN1–pN2 disease, and 55% for pN3 disease (Fig. 4b). Patients with distant metastases (M1) had a 5-year cumulative incidence of 83%.

The most common histological subtype of penile cancer, usual SCC, had a 5-year cumulative incidence of penile cancer-specific death of 16% (14%, 19%). The cumulative incidence of other common subtypes was 17% (12%, 23%) for basaloid, 8.8% (4.1%, 16%) for verrucous, and 5.7% (1.0%, 17%) for warty subtypes. The histological subtype with the highest cumulative incidence of penile cancer-specific death was the rare clear cell tumors with a cumulative incidence of 54% (20%, 79%).

Time trends in KM survival and cumulative incidence were evaluated for the following subgroups: the overall cohort, patients managed with curative intent, patients with AJCC stage IV disease, M1 disease, pN1, pN3, and pNx status. Although stage IV and M1 disease mortality declined through the time periods, stage IV from 64% (48%, 77%) to 55% (42%, 66%) and M1 disease from 92% (33%, 99%) to 74% (47%, 89%), no statistically significant differences in survival across time periods were observed in any subgroup.

**Fig. 5:**
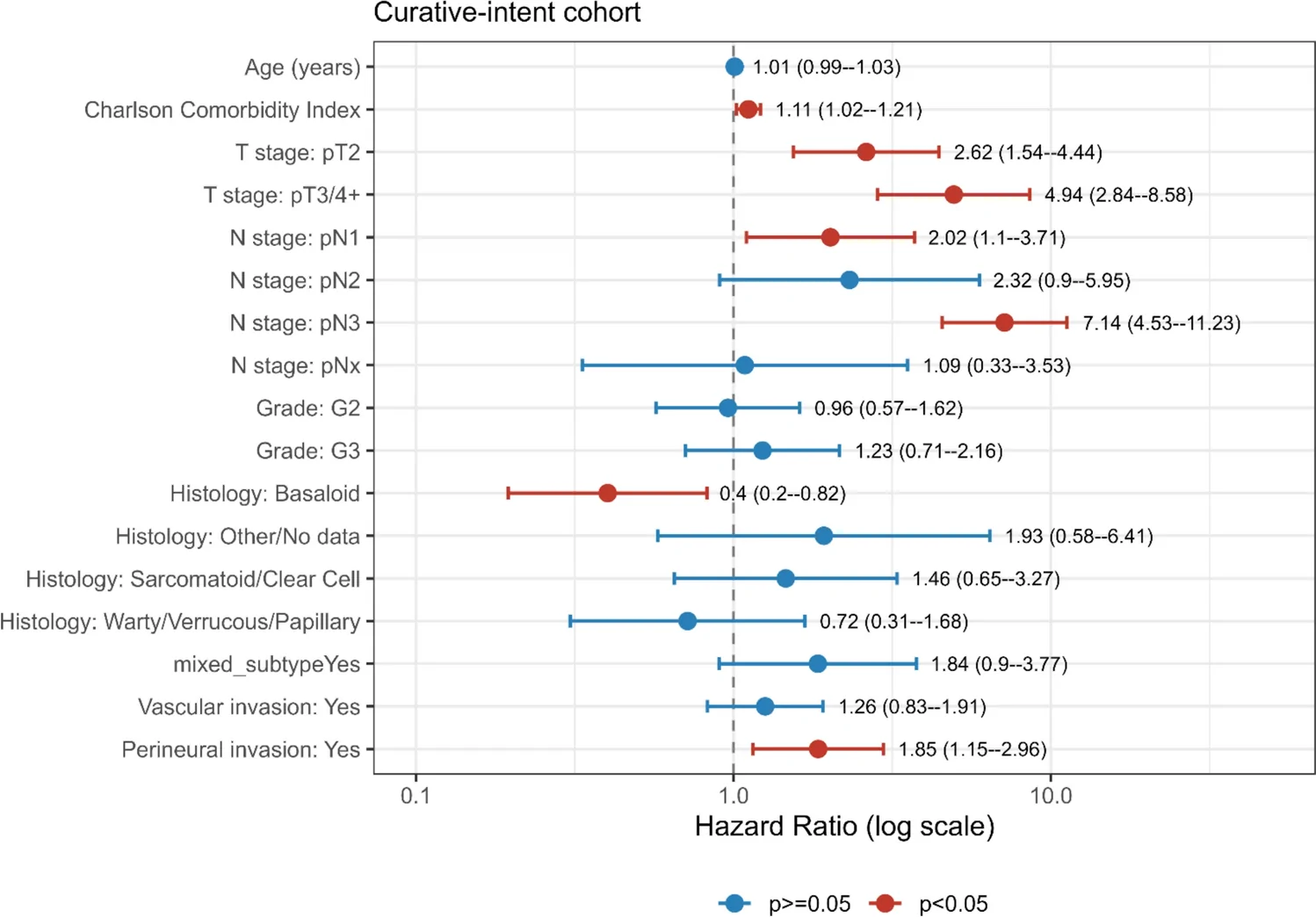
Forest plot of hazard ratios from multivariable Cox proportional hazards regression for disease-specific survival in the curative-intent cohort (*n* = 1200). Hazard ratios are presented with 95% confidence intervals on a log scale. Red indicates statistical significance (*p* < 0.05), blue indicates non-significance. Reference categories are: pT1 (T stage), pN0 (N stage), G1 (grade), SCC usual type (histology), No (mixed subtype), No (vascular and perineural invasion). The Grade: No data’ category is omitted from the figure due to a sparse and unstable estimate but is retained in the model. Violations of the proportional hazards assumption were identified for N stage and vascular invasion (Schoenfeld residuals test *p* < 0.001 and *p* = 0.005, respectively). *CCI* Charlson Comorbidity Index

In multivariable analysis, T stage and N stage were the strongest independent predictors of disease-specific survival (Fig. 5). Compared to pT1, pT2, and pT3/4 + tumors carried 2.6-fold (HR 2.62, 95% CI 1.54–4.44, *p* < 0.001) and 4.9-fold (HR 4.94, 95% CI 2.84–8.58, *p* < 0.001) higher hazards of penile cancer death, respectively. Among nodal stages, pN1 (HR 2.02, 95% CI 1.10–3.71, *p* = 0.024) and pN3 (HR 7.14, 95% CI 4.53–11.23, *p* < 0.001) were significantly associated with worse DSS compared to pN0, while pN2 showed a non-significant trend (HR 2.32, *p* = 0.080) and pNx did not differ from pN0 (HR 1.09, *p* = 0.892). Perineural invasion independently increased the hazard of cancer-specific death (HR 1.85, 95% CI 1.15–2.96, *p* = 0.011), as did comorbidity burden (HR per CCI unit 1.11, 95% CI 1.02–1.21, *p* = 0.016). Basaloid histology was associated with significantly better DSS compared to SCC usual type (HR 0.40, 95% CI 0.20–0.82, *p* = 0.013). Age, grade, mixed subtype, and vascular invasion were not independently significant.

## Discussion

### Strengths and limitations

Penile cancer databases remain scarce due to the rarity of the disease, making it challenging and time consuming to gather large patient cohorts within a single registry. Most larger databases are based solely on national or international registries, or administrative codes. In contrast, the DaPeCa-data database is a clinical database constructed from collected data from medical and pathology charts, providing a more granular and comprehensive dataset. This clinical foundation makes the DaPeCa-data database particularly valuable for evaluating clinically relevant associations and tracking development in the diagnosis and management of penile cancer in Denmark.

However, the retrospective design inherently carries limitations. Certain variables in the DaPeCa-data database—such as the Charlson Comorbidity Index and living arrangement—may be subject to reporting bias. Additionally, as we did not have access to all patient records from hospitals outside the two participating centers, clinical events that occurred in other Danish regions or at the patient’s general practitioner could not be captured. This limitation is particularly relevant when evaluating outcomes such as cause of death, which may be difficult to determine accurately if the patient received end-of-life care outside the catchment area of our database.

Additionally, a proportion of cases likely remain undiagnosed. Some patients with microinvasive disease may be treated with minor procedures such as excision, local resection, or laser ablation, for example, in private dermatology practice—often without histopathological confirmation—thereby escaping formal registration.

### Cancer characteristics

We found a TNM stage distribution comparable to that reported in other European populations [6–11].

The 13% rate of undetermined nodal status (pNx) in our cohort likely reflects clinical reality in a population-based series spanning 26 years rather than systematic understaging. Patients with pNx were older, more comorbid, and had poorer performance status than staged patients, suggesting a group often unfit for sentinel node biopsy or lymph node dissection; accordingly, 68% were managed with palliative intent. Among the remaining 32% treated with curative intent, omission of formal nodal staging may reflect alternative clinical pathways—for example, patients with radiologically evident bulky inguinal disease or distant metastases proceeding directly to oncological therapy. The lack of independent prognostic impact of pNx after adjustment for confounders supports this interpretation. Nevertheless, some curatively treated pNx patients may harbor occult nodal metastases. Efforts to minimize pNx in surgically fit patients remain important, as nodal status is the strongest determinant of survival in penile cancer.

In our study, we observed a modest shift toward more advanced disease over the study period. A similar trend was noted in histopathological grade and tumor subtype, with more aggressive histological variants comprising a greater proportion of cases in the more recent cohorts. Metastasis (M) stage was stable throughout the study period. We do not believe that this pattern of increasingly advanced disease suggests that penile cancer in Denmark is becoming a more aggressive disease at presentation.

We think it is a referral phenomenon. We believe that a larger proportion of penile cancer patients is now referred to specialized centers for evaluation, a group of patient with extensive disease who were more frequently left to palliative strategies in non-specialized settings at local and regional hospitals in earlier years are now referred to specialized centers and into the database.

Other possible explanations include delayed help-seeking behavior, potentially leading to diagnosis at more advanced stages, or improved diagnostic accuracy over time. The latter may reflect advances in staging practices, greater detection of nodal and distant metastases, and increased involvement of specialized pathologists with expertise in penile cancer. It is also possible that a higher proportion of patients with advanced disease are now reaching hospital care, whereas some may have remained undiagnosed in earlier periods. Supporting this interpretation, we observed an increasing proportion of patients managed with palliative intent over time.

### Age

The median age of our cohort is 68 years at the time of diagnosis, with a subtle shift towards more elderly patients in more recent years. This finding is consistent with studies from other high-income countries, where the reported age at diagnosis typically ranges from 65 to 71 years [6, 8–10].

Although lower survival estimates have been observed among younger patients in previous studies [12, 13], we found that the youngest patients in our cohort had the best disease-specific survival. However, this observation is based on a relatively small sample size of 22 men under the age of 40 and should be interpreted with caution. Systematic and publicly re-imbursed HPV vaccination of Danish boys was not offered until 2019 and thus we do not expect HPV vaccination to significantly influence the current dataset.

### Treatment

Penile cancer treatment decisions have significant implications for sexual function, quality of life, body image, and gender identity. Balancing oncologic control with penile preservation remains a clinical challenge. While many studies have addressed this issue [14–16], the rarity of penile cancer limits the availability of large, methodologically robust cohorts, making definitive evidence on the oncologic safety of OPT versus partial or total penectomy difficult to establish. Nonetheless, the literature consistently supports an individualized approach for T1 and T2 tumors, considering tumor aggressiveness while aiming to preserve function and optimize survivorship.

The observed shift away from penile surgery alone towards combined penile and lymph node surgery over the study period likely reflects the increasing adoption of sentinel node biopsy and systematic lymph node staging in Danish penile cancer management. In Demark, SNB has been implemented as golden standard for all penile cancer patients throughout the shole study period, but the change after 2009 from 5 to 2 specialized tertiary centers might be the cause behind the drop in patients not undergoing other treatment than penile surgery. The low and stable proportion of patients receiving oncological treatment without lymph node surgery suggests that oncologic treatment as a primary modality remains uncommon in the curative-intent setting. The shift towards adjuvant chemoradiation in more recent periods aligns with accumulating evidence supporting the superiority of platinum-based chemoradiation over radiotherapy alone in locoregionally advanced penile cancer [17].

As expected, treatment modality differed markedly by stage. The high proportion of stage III–IV patients receiving surgery combined with chemoradiation reflects the aggressive multimodal approach applied to locoregionally advanced disease, while the 4% receiving oncological treatment without lymph node surgery may represent patients deemed inoperable at presentation or those with rapidly progressive disease precluding surgical staging.

We found an increase in initial non-curative or palliative primary tumor treatment strategy over time. This could be indicative of an increase in frail patients in the cohort—as suggested by the shift toward higher CCI scores and age over the study period—or of the shift towards slightly more advanced tumor stages as time progressed. Additionally, it may mirror a broader tendency seen in most surgical specialties to more cautiously balance the potential benefits of curative treatment against the risks of overtreatment in patients who are elderly, comorbid, or otherwise too frail to recover successfully from oncological surgery [18, 19].

### Survival

Overall, this study demonstrates non-inferior survival outcomes compared with those reported from neighboring countries and previously published population-based data worldwide (6, 7, 20–23). These findings may, at least in part, be attributable to the centralized and highly specialized management of penile cancer in Denmark, including routine surgical nodal staging with SNB recommended for all patients, resulting in a high level of histological nodal staging.

Conversely, as the database is not derived from pathology registries, patients with advanced disease managed outside the catchment area or not referred to a tertiary penile cancer center may be underrepresented, potentially leading to an overestimation of survival outcomes. We believe this to be a minor issue informed by DaPeCa-audits done twice since 2011 on this aspect.

We presented our latest update of our national database on Danish penile cancer patients and their prognosis which appears comparable to other countries with lymph node evaluation at the time of penile surgical treatment.

## Conclusion

This study presents one of the largest national clinical databases on penile cancer, with near-complete surgical tumor staging and a high rate of surgical nodal staging. Covering 26 years of clinically curated data, the database provides a robust foundation for evaluating penile cancer management and outcomes. Now maintained as a permanent prospective platform, it will continue to support future research and quality improvement.

## Data Availability

The data that support the findings of this study are not publicly available due to Danish data protection legislation and regulations governing access to national health registry data. Data are available from the authors upon reasonable request and with permission from the relevant Danish authorities.
